# Super Hydrophobic UHMWPE/PTFE/PVA Composites with Low Friction: Preparation and Wear Mechanism

**DOI:** 10.3390/polym17121664

**Published:** 2025-06-16

**Authors:** Hai Wang, Zhiwei Shao, Kuiyuan Shen, Buhe Bateer, Fushen Ren, Xiaowen Qi

**Affiliations:** 1 Bohai Rim Energy Research Institute, Northeast Petroleum University, Qinhuangdao 066004, China; wh4019@nepu.edu.cn (H.W.); renfushen@126.com (F.R.); 2 Department Mechanical Science and Engineering, Northeast Petroleum University, Daqing 163318, China; szw2496507244@163.com (Z.S.); 15890181604@163.com (K.S.); 3 Aviation Key Laboratory of Science and Technology on Generic Technology of Self-Lubricating Spherical Plain Bearing, Yanshan University, Qinhuangdao 066004, China

**Keywords:** UHMWPE, PTFE, PVA, tribological properties, wear mechanism

## Abstract

This study develops novel superhydrophobic UHMWPE/PTFE/PVA composites via hot-pressing sintering to achieve ultra-low friction and enhanced wear resistance. The ternary system synergistically combines UHMWPE’s mechanical stability, PTFE’s lubricity, and PVA’s dispersion/binding capability. Results show PTFE disrupts UHMWPE crystallization, reducing melting temperature by 2.77 °C and enabling energy dissipation. All composites exhibit hydrophobicity, with optimal formulations (UPP3/UPP4) reaching superhydrophobicity. Tribological testing under varied loads and frequencies reveals low friction, where UPP1 achieves a COF of 0.043 and wear rate below 1.5 × 10^−5^ mm^3^/(N·m) under low-load conditions. UHMWPE oxidative degradation forming carboxylic acids at the interface (C=O at 289 eV, C–O at 286 eV). Formation of tungsten oxides (WO_3_/WO_2_), carbides (WC), and transfer films on steel counterparts. A four-step tribochemical reaction pathway is established. PVA promotes uniform transfer films, while PTFE lamellar peeling and UHMWPE chain stability enable sustained lubrication. Carbon-rich stratified accumulations under high-load/speed increase COF via abrasive effects. The composites demonstrate exceptional biocompatibility and provide a scalable solution for biomedical and industrial tribological applications.

## 1. Introduction

Self-lubricating polymer composites play indispensable roles in biomedical implants, automotive engineering, and aerospace applications due to their low friction coefficients, high wear resistance, and corrosion stability [1,2]. Among these, UHMWPE stands out for its exceptional impact strength, chemical inertness, biocompatibility, and intrinsically low friction coefficient [3,4], making it ideal for articular prostheses and bearing components. However, unmodified UHMWPE suffers from prohibitively high wear rates, where generated debris can cause osteolysis and implant failure in biomedical contexts [4] or compromise component longevity in industrial settings. While modification strategies—including reinforcement phases (e.g., basalt fibers [5], carbon fibers [6,7]), lubricating fillers (e.g., PTFE [8], MoS_2_ [9]), and surface treatments [10,11]—have shown promise, critical limitations persist: most approaches sacrifice either wear resistance or friction reduction rather than optimizing both synergistically [12,13,14]; The wear depth was reduced by approximately 14%. UHMWPE/PTFE composites with different PTFE contents (5–40 wt.%) were prepared by hot pressing method, and 10 wt.% PTFE was determined to be the best content. But PTFE exhibits poor interfacial adhesion with UHMWPE, leading to discontinuous transfer films [8]. Chukov et al. [15] further studied and found that the wear loss of 8 wt.% micro carbon fiber composite was 50% lower than that of pure UHMWPE. The friction coefficient of UHMWPE fiber/UHMWPE powder blend composite is lower than that of pure UHMWPE, and the tribological performance is the best when 50% is added [16]. The synergistic effect of UHMWPE/CF/MoS_2_ has a low coefficient of friction (0.11–0.15) and wear rate (3.8–7.6 × 10^−5^ mm^3^/N·m). With the progress of friction, the wear mechanism changes from abrasive wear to fatigue wear [17]. The biomimetic composite material composed of natural chia seed polysaccharides and UHMWPE attracts water molecules through non-covalent interactions under water conditions, forming a gel layer at the friction interface and achieving a relatively low coefficient of friction [18]. The tribological properties of UHMWPE composites are improved by using natural mineral wollastonite as a filler. Under the conditions of dry friction, normal saline lubricant and serum lubricant of newborn calves, the wear rates decreased by 71%, 69.81% and 50.73% respectively. The main wear mechanisms are slight fatigue wear and abrasive wear [19]. UHMWPE filled with 90-degree oriented graphene has good thermal conductivity and a stable structure. Compared with pure UHMWPE, the wear rate of UHMWPE with 90-degree graphene added is reduced to 97.29% [20]. The UHMWPE lubricating layer was interlocked in the 3D-printed polyetheretherketone (PEEK) scaffold, and hyaluronic acid was introduced to modify the hydrophilicity of the UHMWPE matrix. The HA embedded in the substrate can continuously appear on the friction interface, firmly forming a boundary lubrication layer, thereby enhancing the lubrication performance of the UHMWPE component. Under the equivalent joint load and low friction speed, the friction coefficient of the support can be as low as 0.041 [21].

However, the combined effects of rigid reinforcers and solid lubricants on tribochemical mechanisms remain underexplored; and the impact of fabrication processes like hot-pressing sintering on multi-component composites is poorly understood. To address these gaps, this work develops a novel ternary composite system—combining UHMWPE as the matrix, PTFE as the lubricating phase, and PVA as the reinforcing phase—fabricated via hot-pressing sintering. We aim to elucidate the synergistic mechanism enabling low friction and high hydrophobicity, characterize wear mechanisms and transfer film formation through interfacial tribochemical analysis and establish a scalable processing framework for high-performance UHMWPE composites in tribological applications, especially in biotribology.

## 2. Materials and Methods

### 2.1. Materials

UHMWPE and P-ASP were provided by Tech-in Materials Co., Ltd. (Nanjing, China). The average diameters were 30 μm and 1 mm, respectively. PTFE in a particle size of 500 nm was supplied in powder form by DAIKIN fluorochemicals (Changshu, China) Co., Ltd. The mass ratio of raw materials for preparing UPPs is shown in Table 1. PVA adopts PVA1788 (Macklin, Shanghai, China), with a low degree of polymerization, partially alcoholized type, and a molecular weight of 20,000.

**Figure 1:**
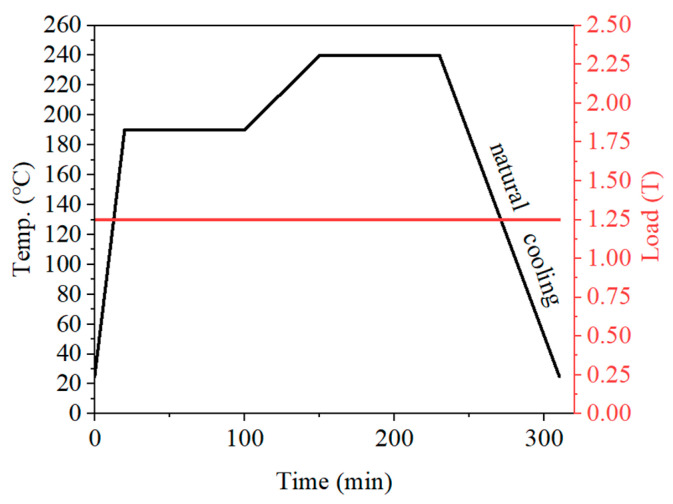
The hot-pressing sintering process curve of UPPs.

The hot-pressing sintering process consists of two steps: solution mixing and hot-pressing sintering. Mix the ultra-high molecular weight polyethylene powder, polytetrafluoroethylene particles and polyvinyl alcohol powder in the proportion shown in Table 1 simultaneously through ultrasonic vibration and mechanical stirring for 2 h (as PVA1788 has a low degree of polymerization, the stirring time is longer), and then place them in a drying oven for use. The dried UPPs sample powder was taken out for vacuum filtration and cleaning. This process was repeated three times. The washed UPPs sample powder was placed in a forced convection drying oven at 80 degrees Celsius for drying for 24 h. The dried UPPs sample powder was formed at room temperature through a hot press mold. The heating and pressure curves are shown in Figure 1 (The black line represents the temperature and the red line represents the loading force).

### 2.2. Experimental Characterization and Testing

Rockwell hardness indenter (Diameter: 6.35 mm) was used to evaluate the hardness of UPPs according to ISO ISO 3290:2014 standard [22]. An electronic solid density meter (MDJ-120TS, Gehrden, Germany) was utilized to determine the material density. Before tribological test, UPPs were polished using 1000# abrasive papers (Ra = 10 μm), as well as ultrasonically cleaned with absolute alcohol for 30 min. A ball-on-disk tribometer (CSM3, Peseux, Austria) were used to test the coefficient of friction (COF). The upper specimen wasYG6 ball (ISO: G5) with the diameter of 6 mm. Because of the light weight and good tribological properties, UPPs can be used in applications that do not require large mass loads. For example, some sliding pairs under non-bearing and normal temperature condition on CNC machine tools. The test conditions were shown in Table 2. Each test was repeated at least three times to warrant the test reproducibility with reported average data and associated standard deviations. The contact load was calculated using the Hertzian contact stress formula with the elastic modulus and Poisson’s ratio of pure UHMWPE as input parameters.

The wear morphology and cross-sectional area of UPPs were obtained by MicroXAM-800 (KLA-Tencor, Milpitas, CA, USA). The cross-sectional areas at 4 different positions were chosen to calculate the average data according to the following formulae:

ΔV = Sd(1)

W = ΔV/(F × L)(2)

where W is the wear rate (mm^3^/N·m), S is the mean cross-sectional area of wear track (μm^2^), d is the length of wear track (mm), F is a test load (N) and L is the sliding distance (mm).

The scanning electron microscope (SEM, JSM-6701F, Tokyo, Japan) attached with energy dispersive X-ray spectroscopy (EDS) was employed to examine morphological structures of worn surfaces, as well as the element distribution of transfer films. The decomposition temperature of serpentine powders was investigated by a thermal analyzer (Hitachi DSC–200, Akishima, Japan) which allows simultaneous differential scanning calorimetry (DSC) analysis. The range of scanning temperature was chosen from room temperature to 1000 °C, while the heating rate was at 10 °C/min. The protection gas was argon gas. The crystallinity calculation Formula (3) of UPPS is shown as follows.

(3) $$X_{c}=\frac{{∆H}_{m}}{{∆H}_{m}^{0}}\times 100\%$$

${∆H}_{m}$ is sample melting enthalpy (by DSC curve is melting peak area), ${∆H}_{m}^{0}$ is completely crystallization the melting enthalpy of the sample.

X-ray photoelectron spectroscopy (XPS) was conducted on a Thermo ScientificTM K-AlphaTM+ spectrometer (Thermo Fisher Scientific, Waltham, MA, USA) equipped with a monochromatic Al Kα X-ray source (1486.6 eV) operating at 100 W.

## 3. Results

### 3.1. Physical Properties

**Figure 2:**
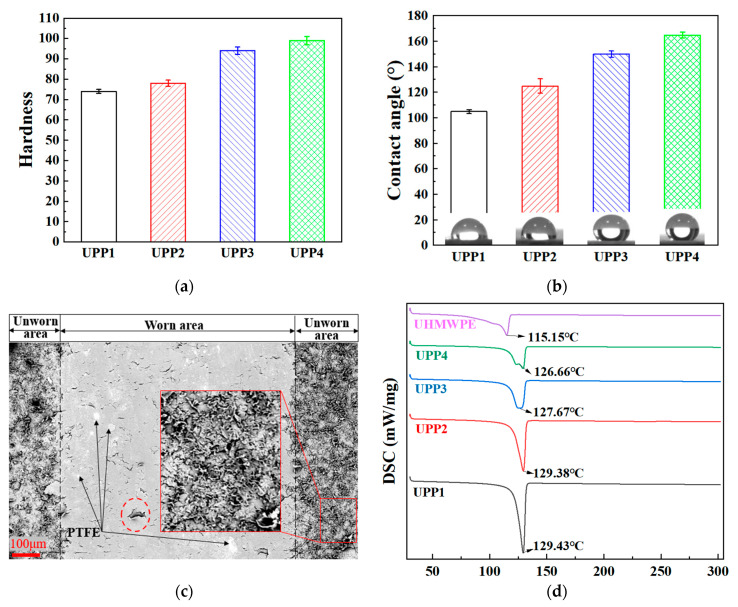
(**a**) Hardness, (**b**) contact angles, (**c**) surface topography of UPPs, (**d**) DSC.

The hardness and contact angles of UPPs are depicted in Figure 2a,b. With the increase of PTFE mass proportion, the hardness and contact angle of UPPs increase monotonically. The hardness of pure UHMWPE and pure PTFE is both below 65, while the hardness of UPPs is higher than 70 HRB, the hardness of UPP3 and UPP4 is even more than 90 HRB. The increase of hardness can enhance the mechanical properties. The higher the contact angle of UPPs, the stronger the hydrophobicity and the lower the surface energy, resulting in a smaller real contact area, which is conducive to reducing friction. The dashed lines in Figure 2c show the morphology of the unworn region of the UPP showing a typical microstructural superhydrophobic morphology [23], as shown in the partial enlargement. The worn area has been removed from the surface topography of the superhydrophobic physical microstructure, and the typical PTFE distribution can be clearly seen, as indicated by arrows. In Figure 2c, an obvious layered structure appears in the dashed line frame. The red wireframe enlarged image presents the typical surface morphology of the physical superhydrophobic structure. PTFE is very prone to lamellar peeling, and PVA acts as adherend, so the wear area is very flat because a lubricating film has been generated.

The DSC thermograms of UPPs demonstrate distinct endothermic peaks corresponding to polymer melting (Figure 2d). The melting peak of pure UHMWPE is 115.15 °C, and the initial temperature of the peak is 103.69 °C. For the 1% sample, an endothermic peak with an onset temperature of 122.1 °C and peak temperature of 129.43 °C is observed. The 3% sample exhibits a similar melting peak at 129.38 °C with an onset temperature of 120.62 °C. Notably, both the onset and peak temperatures progressively decrease with increasing PTFE content: the 5% sample shows a peak at 127.67 °C (onset 117.20 °C), while the 7% sample displays a peak at 126.66 °C (onset 116.65 °C). Concurrently, the integrated area of the endothermic peaks (representing melting enthalpy) diminishes with higher PTFE concentrations. This thermal behavior can be attributed to PTFE’s influence on UHMWPE crystallization. The calculation results based on Table 1 and Figure 2d are shown in Table 3.

As can be seen from Table 3, with the increase of PTFE content, the crystallinity of UPP gradually decreases. When the proportion of PTFE reaches 34.8%, the crystallinity increases instead, which may be caused by the high PTFE content hindering phase separation. PTFE modifies the crystallization dynamics through several mechanisms: (1) Its molecular structure disrupts UHMWPE’s crystalline organization, reducing structural compactness; (2) Weak interfacial interactions between PTFE and UHMWPE enhance chain mobility within the polymer matrix; (3) PTFE particles act as physical barriers during crystal growth, restricting crystalline domain development. These combined effects lower the energy required for crystal melting, thereby decreasing both the onset and peak melting temperatures. The observed reduction in melting enthalpy further confirms PTFE’s role in impairing the formation of well-ordered crystalline structures in UHMWPE. The reduced crystallinity induced by PTFE incorporation further enhances molecular chain mobility, facilitating energy dissipation via viscoelastic deformation rather than frictional heat generation.

### 3.2. Tribological Properties

**Figure 3:**
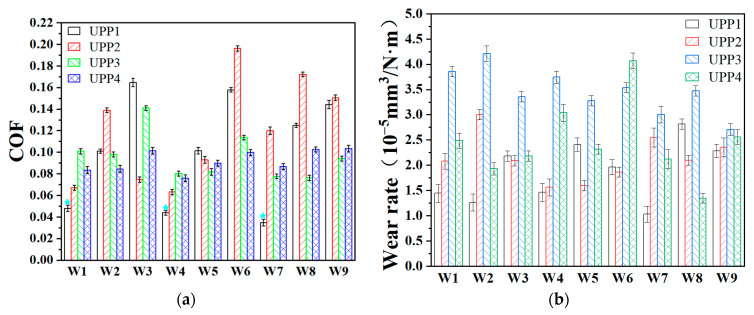
(**a**) COFs and (**b**) wear rate of UPPs.

Figure 3a demonstrates the COFs and wear rate (Figure 3b) of UPPs.

The coefficient of friction (COF) of UPPs exhibited a trend of initially increasing and then decreasing with increasing PTFE content. Under low testing speeds, a lower PTFE content proved sufficient to facilitate the formation and spreading of transfer films. As the testing speed increased, intensified wear generated more PTFE debris particles, which underwent processes of exfoliation, transfer, and ultimately adhesion to the counterface steel ball to form a transfer film. Moreover, both elevated testing loads and speeds resulted in an overall increase in COF across all UPPs compositions. However, consistent with previous studies [24,25], an optimal PTFE content range existed for achieving minimal COF, rather than a monotonic decrease with higher PTFE content. Notably, the UPPs materials demonstrated exceptionally low friction characteristics, with all four formulations maintaining COF values below 0.2 under conditions W1–W9. Particularly, UPP1 exhibited ultra-low COF values (0.043) under W1, W4, and W7 conditions.

Although PTFE typically serves as a friction-reducing phase in composites, it demonstrates poor wear resistance. Consequently, UPP1 (with minimal PTFE content) exhibited the lowest wear rate under low-load and low-speed conditions. The wear rate of the composites increased progressively with higher PTFE content. Nevertheless, consistent with the friction behavior, an optimal composition ratio yielded the minimum wear rate. Specifically, UPP1 demonstrated superior wear resistance with wear rates below 1.5 × 10^−5^ mm^3^/(N·m) under W1, W2, W4, and W7 testing conditions.

**Figure 4:**
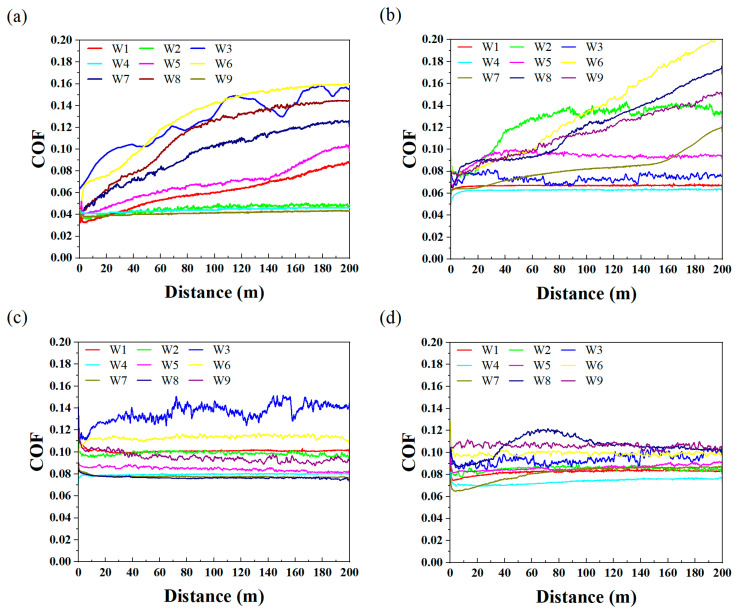
Single group COFs at the condition (**a**) U1, (**b**) U2, (**c**) U3, (**d**) U4.

Figure 4 illustrates the single group COFs of various UPPs. Only UPP2 exhibited a friction coefficient of 0.2 after 200 m of sliding distance, with a continuing upward trend. The other three UPPs maintained stable and relatively low friction coefficients. UPP4 demonstrated exceptional performance with friction coefficients consistently below 0.12 under all tested conditions. Comprehensive analysis of the four datasets revealed that elevated friction coefficients predominantly occurred under high-load or high-frequency conditions, attributed to the disruption of the dynamic equilibrium in transfer films under severe operational environments. The running-in periods of UPP3 and UPP4 were remarkably short, accompanied by low coefficient of friction (COF) values and minimal fluctuation amplitudes in their friction curves. This behavior demonstrates excellent material consistency, further evidenced by their stable tribological performance. These characteristics highlight their significant potential to serve as competitive alternatives to metallic materials in tribological applications.

### 3.3. Wear Morphology

**Figure 5:**
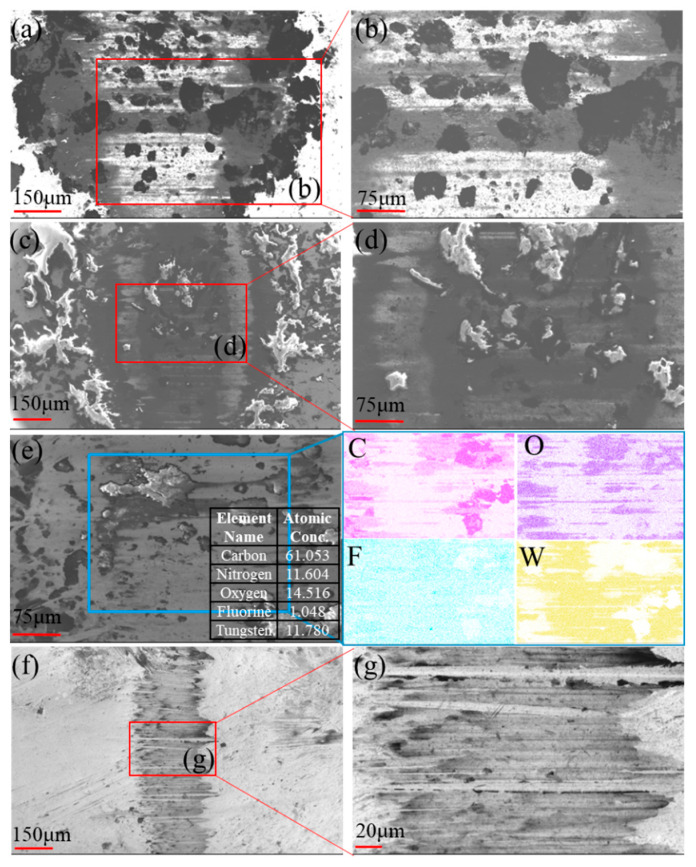
Surface morphology of YG6 ball: (**a**,**b**) 10 N-1 Hz, (**c**,**d**) 10 N-4 Hz, (**e**) 2 N-2 Hz and (**f**,**g**) 2 N-1 Hz.

Figure 5 is SEM images of the friction contact area of YG6 ball sliding against UPP1. Under high-load experimental conditions, distinct stratified accumulations were observed on the steel ball surface (Figure 5a,b). Carbon-rich accumulations were uniformly distributed at both the edge and center of the wear scar [26], indicating their continuous generation and inability to discharge from the friction interface during operation. The blocky carbon-rich products covering the light-colored transfer film directly contacted the steel ball, resulting in a high friction coefficient. Similarly, stratified accumulations emerged at elevated sliding speeds. However, the increased speed introduced more oxygen into the friction process, transforming these accumulations from carbon-rich products to carbon-oxygen compounds. These substances showed similar matrix coloration to the original UPP wear debris, suggesting no chemical structural alteration. These accumulations likely originated from the lubricating layer formed through frictional interaction of UHMWPE, polyvinyl alcohol, and PTFE (Figure 2c), which subsequently transferred to the steel surface. Despite the friction coefficient reduction compared to high-load conditions due to the stratified structure (Figure 5c,d) that significantly decreased actual contact area, the value remained higher than that under light-load scenarios.

When the load decreased from 10 N to 2 N, minimal stratified accumulations persisted on the steel surface (Figure 5e). EDS analysis revealed uniform fluorine distribution across most wear regions, indicating formation of an ultrathin transfer film. This effective film coverage with limited multilayer accumulation accounted for the substantial friction coefficient reduction [27] and low wear rate of UPP1 (Figure 3b). Further frequency reduction dramatically minimized the wear scar dimensions (Figure 5f). A narrow wear track with uniform thick transfer film coverage was observed (Figure 5g). This high-quality transfer film contributed to the lowest friction coefficient under low-load/low-speed conditions. Although the transfer film exhibited smaller coverage area, its uniform distribution and greater thickness resulted in slightly higher wear rate compared to high-speed conditions (Figure 3b).

## 4. Discussion

**Figure 6:**
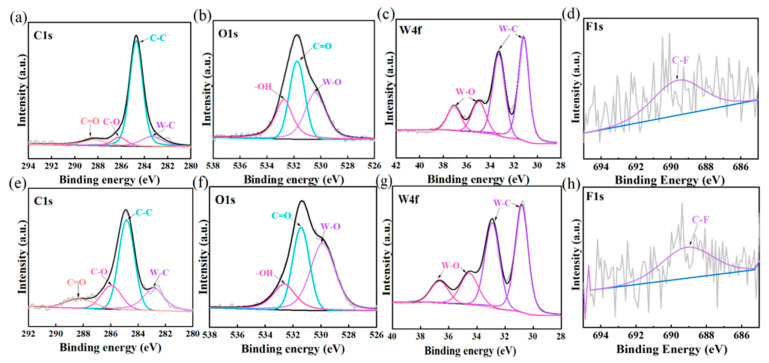
XPS spectral analysis of the worn surface of YG6 balls: (**a**–**d**) UPP1, (**e**–**h**) UPP3.

The transfer film and carbide deposits formed in the wear area of the steel ball prevent the direct contact between UPPs and the metal surface, thereby reducing the coefficient of friction, as shown in Figure 6.

It is difficult to confirm the chemical changes during the friction and wear process only through rate morphology analysis, so XPS assistance is usually required. In the C1s spectra of Figure 6a,e, the peak at 289 eV is the C=O characteristic peak, which comes from the carboxylic acid carbonyl carbon [28]. The C-O bond at the 286 eV position is the characteristic peak of the carboxyl group in carboxylic acids [29]. In the O1s spectra of Figure 6b,f, the peak at 531 eV is the characteristic peak of C=O, and the -OH at 533 eV is the typical hydrogen peroxide peak of carboxylic acid. The -OH characteristic peak located at 532.5 eV in the O1s spectra of Figure 6b,f is derived from the carbonyl oxygen of the carboxylic acid. At 530 eV is the characteristic peak of W-O, which proves that perhaps WO3 is generated. In Figure 6c,g, the typical oxidized state of WO_3_ (W^6+^) appears at 35–38 eV, with a characteristic peak structure (4f_7_/_2_ and 4f_5_/_2_, with an interval of approximately 2.1 eV and an intensity ratio of 4:3). The W-O characteristic peak in Figure 6g is slightly shifted. The W 4f peak shifts towards the direction of high binding energy. The W-C bond combined with the C 1s peak at 283.5 eV may point to the presence of tungsten carbide. The high carbon content in Figure 5e also proves this point [30]. Moreover, the W 4f_7_/_2_ peak at 33–34 eV indicates the presence of tungsten oxide WO_2_ [31]. Finally, the C-F characteristic peaks in Figure 6d,h indicate the existence of the transfer membrane [32]. To sum up, it indicates that the oxidation of UHMWPE to form carboxylic acid occurs at the friction interface. The specific process is as follows:

(i) Frictional stress causes the molecular chain of UHMWPE to break, generating alkyl radicals (R•).

$$–CH_{2}–CH_{2}–\;\overset{shear}{\to }\;–CH_{2}\cdot +–CH_{2}\cdot$$

(ii) Free radicals react with oxygen (O_2_) or water (H_2_O) to form peroxide intermediates:

$$R–CH_{2}\cdot +O_{2}\to R–CH_{2}–O–O$$

(iii) Peroxyl radicals further decompose into aldehydes (R-CHO) or ketones (R-CO-R’):

$$R–CH_{2}–O–O\cdot \to R+CHO+HO$$

(iv) Aldehydes are oxidized to carboxylic acids (R-COOH) in the presence of oxygen and water:

$$R–CHO+H_{2}O+O_{2}\overset{oxidation}{\to }R–COOH+H_{2}O_{2}$$

Figure 6 confirmed that the oxidative degradation of UHMWPE occurred at the friction interface, generating products containing carboxylic acid groups (Figure 6a,b,e,f), and it was detected that a complex layer containing tungsten oxides (WO_3_, WO_2_), carbides (Figure 6c,g), and fluorine-containing transfer films (C-F bonds) was formed on the worn surface of the steel balls. These findings support the proposed reaction mechanism, especially the final formation of carboxylic acid (iv) and the oxidation/carbonization occurring on the metal surface. Figure 6d,h confirms that a transfer film is formed on the surface of the steel balls because pure UHMWPE does not contain the F element.

The high molecular weight and crystallinity of UHMWPE endow it with excellent wear resistance, which can form a more stable mechanical support layer during friction, reducing the rupture and detachment of PTFE transfer films and thus prolonging the lubrication effect. Under the action of frictional heat and oxygen, UHMWPE may undergo oxidative degradation, generating carboxylic acids. Its ultra-long molecular chain can initiate cross-linking through mechanical action. Form a denser surface structure to inhibit further wear. XPS demonstrated that the sharp edges of the formed tungsten carbide particles might scratch the polymer matrix, resulting in a higher wear rate.

Because UHMWPE is often used in the biomedical field, the materials selected in this study have good biocompatibility. Among them, PTFE is one of the common materials of bio-lubricating materials, and the reason for its low friction coefficient is that it is easy to flake off, which is very conducive to the formation of transfer film when PTFE is used as a filler [33]. PVA can be degraded by microorganisms in the natural environment (subject to specific conditions) and is non-toxic to humans [34]. The hydrophilic and steric hindrance effects of PVA can prevent particles from agglomerating and improve the uniform dispersion of components during the preparation of composite materials [35]. At the same time, PVA itself has good film formation, and a small amount of PVA plays the role of binder to promote the rapid formation of transfer film.

**Figure 7:**
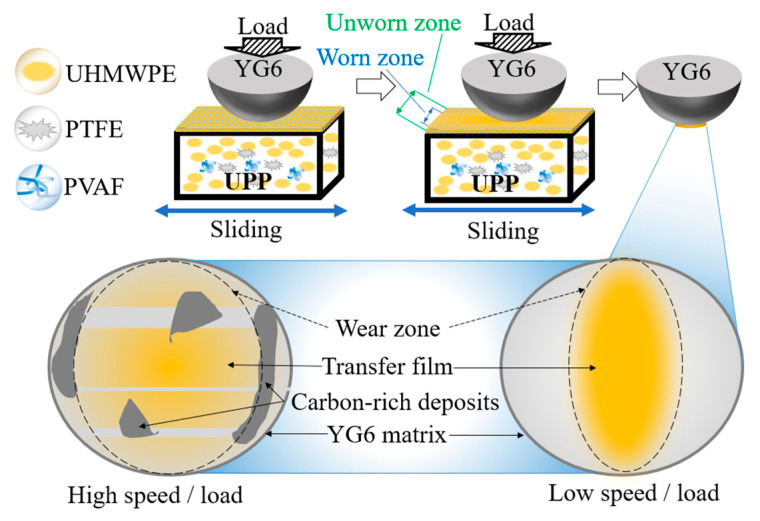
Schematic diagram of the friction mechanism.

Figure 7 shows the schematic diagram of friction mechanism of UPPs. During the friction process, the frictional cutting effect of the steel ball on the UPPs surface material disrupted the superhydrophobic microstructure, and the morphology of the wear mark area changed. Then, part of the surface material is peeled off and transferred to the surface of the steel balls of the friction pair, forming a transfer film, and the friction coefficient decreases, entering the stable period. At this point, the interfacial friction changes from “UPP-YG6” to “UPP-transfer film”, maintaining a low coefficient of friction. Under low-speed or low-load conditions, a thin polymer transfer film covering the surface of the steel balls is formed. Under high-speed or high-load conditions, the stripped surface material fails to fully spread and form a transfer film during the process of being transferred to the surface of the steel ball, and adheres to the surface of the steel ball under the effect of frictional temperature rise. As more and more materials are transferred by friction and shearing, these carbon-rich adherend gradually become larger and thicker, eventually forming carbon-rich zones. The surface smoothness drops sharply, the friction force increases, and the friction coefficient is higher than that under low-speed and low-load conditions. These carbon-rich accumulation areas are large in area, uneven in thickness and high in hardness, like “hard particles” scraping UPP, resulting in a high wear rate.

## 5. Conclusions

This study successfully fabricates ternary composites (UPPs) via hot-pressing sintering, combining UHMWPE matrix, PTFE lubricant, and PVA reinforcement. Key outcomes demonstrate:

(1) PTFE disrupts UHMWPE crystallization, reducing melting temperature by up to 2.77 °C and enhancing energy dissipation; all composites exhibit hydrophobicity, with UPP3/UPP4 achieving superhydrophobicity (contact angle > 150°).
(2) UPPs deliver ultra-low friction (COF < 0.2), where UPP1 reaches a minimum COF of 0.043 and wear rate below 1.5 × 10^−5^ mm^3^/(N·m) under low-load/speed conditions.
(3) XPS validates UHMWPE oxidative degradation forming carboxylic acids, alongside tungsten oxides, carbides and PTFE transfer films on steel counterparts. A four-step reaction pathway (chain scission → radical oxidation → aldehyde conversion → carboxylic acid) is established.
(4) Synergistic mechanisms are uncovered: PVA improves dispersion/binding for rapid transfer films; PTFE lamellar peeling enables lubrication; UHMWPE stabilizes mechanical support. Carbon-rich stratified accumulations under high-load/speed increase COF via abrasive “hard particle” effects.

This work provides a scalable fabrication strategy for UHMWPE-based materials balancing ultra-low friction, superhydrophobicity, and wear resistance, with significant potential in biomedical implants and industrial tribology.

## Figures and Tables

**Table 1:** Sample numbers and weight of each component.

	UHMWPE	PVA	PTFE
UPP1	8 g	1 g	1 g
UPP2	10 g	1 g	3 g
UPP3	12 g	1 g	5 g
UPP4	14 g	1 g	8 g

**Table 2:** Description of friction test operating conditions.

	Load	Velocity	Distance
W1	2 N	1 Hz	200 m
W2	2 N	2 Hz	
W3	2 N	4 Hz	
W4	5 N	1 Hz	
W5	5 N	2 Hz	
W6	5 N	4 Hz	
W7	10 N	1 Hz	
W8	10 N	2 Hz	
W9	10 N	4 Hz	

**Table 3:** The degree of crystallinity of UPPs.

	UHMWPE (Prop)	PVA (Prop)	PTFE (Prop)	ΔH/(J/g)	ΔH/(J/g)	X_C_/%
Pure	1.000	0.000	0.000	293.000	195.315	66.660
UPP1	0.800	0.100	0.100	263.400	161.731	61.401
UPP2	0.714	0.071	0.214	247.143	133.925	54.189
UPP3	0.667	0.056	0.278	238.111	105.048	44.117
UPP4	0.609	0.043	0.348	227.478	124.849	54.884

## Data Availability

Data are contained within the article.
